# Factors affecting one-leg standing time in patients with end-stage knee osteoarthritis and the age-related recovery process following total knee arthroplasty

**DOI:** 10.1186/s13018-017-0522-2

**Published:** 2017-02-01

**Authors:** Kengo Harato, Shu Kobayashi, Iwao Kojima, Aiko Sakurai, Hidenori Tanikawa, Yasuo Niki

**Affiliations:** 1 Department of Orthopedic Surgery, Keio University School of Medicine, 35 Shinanomachi, Shinjukuku, Tokyo, 160-8582 Japan; 2 Department of Physical Therapy, Kawasaki Municipal Kawasaki Hospital, 12-1 Shinkawadouri, Kawasakiku, Kawasaki City, Kanagawa Prefecture 210-0013 Japan; 3 Department of Physical Therapy, International University of Health and Welfare, Mita Hospital, 1-4-3 Mita, Minatoku, 108-8329 Tokyo, Japan; 4 Department of Orthopedic Surgery, Kawasaki Municipal Kawasaki Hospital, 12-1 Shinkawadouri, Kawasakiku, Kawasaki City, Kanagawa Prefecture 210-0013 Japan; 5 Department of Orthopedic Surgery, International University of Health and Welfare, Mita Hospital, 1-4-3 Mita, Minatoku, 108-8329 Tokyo, Japan

**Keywords:** Knee osteoarthritis, One-leg standing, Extension limitation, Recovery process, Total knee arthroplasty

## Abstract

**Background:**

The aims of the present study were to investigate the factors affecting one-leg standing (OLS) time in patients with end-stage knee osteoarthritis (OA) and to clarify the age-related recovery process following total knee arthroplasty (TKA) in the early postoperative period.

**Methods:**

A total of 80 knees of 40 patients with knee OA were enrolled. They were asked to perform relaxed standing on one leg for as long as possible. First, OLS time was measured. Second, age, body mass index, knee flexion angle during (KFA) OLS, femorotibial angle (FTA) during OLS, and a visual analogue scale (VAS) for pain were evaluated. Multiple regression analysis was done to identify the factors affecting OLS time. In addition, the recovery process was compared between older and younger patients after TKA.

**Results:**

A larger KFA during OLS, older age, and larger FTA were significantly associated with shorter OLS time. After TKA, postoperative OLS time in older patients did not improve significantly by postoperative day 20, while the time in younger patients improved significantly from postoperative day 19.

**Conclusions:**

Even if subjective knee pain and KFA during OLS improved, longer rehabilitation was required to improve OLS time in older patients in the early postoperative period.

## Background

End-stage knee osteoarthritis (OA) often limits older patients in performing normal activities of daily living. According to previous reports, symptomatic knee OA is observed in approximately 14.4% of men and 28.4% of women over the age of 45 years, and 87% of knee OA cases are bilateral [1, 2]. Some previous studies indicated that patients with symptomatic knee osteoarthritis would have balance impairments during standing as well as during gait [3, 4]. In addition, the importance of each individual’s locomotive organs has become one of the current topics in recent years. Unfortunately, it has been demonstrated that standing balance is exacerbated in patients with end-stage knee OA, and these facts would lead to an increased risk of falling in such patients [5].

The one-leg standing (OLS) test is a widely used clinical tool to evaluate postural steadiness in the standing position for elderly people. According to previous reports, one-leg standing time was associated with subjects’ age, self-assessment of their health status, body mass index, mortality, and the risk of falls [5–10]. Furthermore, it has also been reported to be helpful in identifying elderly persons at an increased risk of future functional dependence [6]. Falls are one of the major health care concerns for elderly people, which lead to various osteoporotic fractures. Therefore, it is important to clarify the knee condition in older patients with knee OA. Hunt et al. demonstrated that predictors of OLS balance in patients with knee OA were radiographic severity, isometric quadriceps peak torque, number of painful knees, varus angle, and pain intensity [11].

On the other hand, total knee arthroplasty (TKA) has traditionally been performed as an effective treatment for patients with end-stage knee OA, by relieving pain, restoring function, and correcting deformity [12–14]. Therefore, TKA would be effective treatment for patients with a short OLS time. Cho et al. showed that OLS balance in patients with knee OA improved significantly after TKA [15]. However, it is unknown whether TKA is really beneficial for elderly people because the subjects in their study were quite young (mean 61.7 years),

We hypothesized that improvement of OLS time would be slower in older patients than in younger patients after correction of knee deformity by TKA. The aims of this study were to investigate factors affecting OLS time in patients with bilateral end-stage knee OA and to clarify the age-related recovery process following TKA.

## Methods

### Subjects

A total of 80 knees of 40 patients (35 females and 5 males; mean age 74 (range, 60–82) years were enrolled in the current investigation. All knees had varus deformities with radiographic OA of grade 4 severity according to the Kellgren-Lawrence classification [16]. Patients with any symptoms in either the hip or ankle joint were excluded from the study. Patients with rheumatoid arthritis, osteonecrosis, lumbar canal stenosis, and symptomatic low back pain were also excluded.

### Testing procedures

**Fig. 1:**
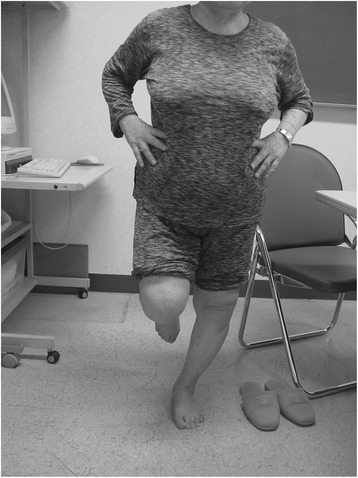
One-leg standing. Subjects were asked to stand on one leg with their eyes open, keeping their arms by their sides

A prospective observational study was conducted at our institution. All subjects provided their informed consent, and the study was approved by our institution. The subjects were asked to stand on one leg for as long as possible without any supportive tools, with their eyes open (Fig. 1). OLS time was measured until the stance foot shifted or the lifted foot was replaced on the floor First, OLS time was measured for each patient, and two trials were done. The better of the two trials was recorded as the OLS time. Second, age, body mass index (BMI), knee flexion angle during one-leg standing (KFA), femorotibial angle (FTA) during one-leg standing, and a visual analogue scale (VAS; 0–100 mm) for pain were evaluated. KFA was assessed using a goniometer, and the FTA was assessed on anteroposterior radiographs of the weight-bearing leg on standing. The FTA was defined as the lateral angle formed by the femur and tibia. Lines were drawn through the middle of the femoral shaft and through the middle of the tibial shaft.

### Operation technique and postoperative evaluation

All patients with knee OA underwent unilateral TKA using the Balanced Knee System®, posterior stabilized design (Ortho Development, Draper, UT) under general and epidural anesthesia. The same technique, including the conventional medial midvastus approach, intramedullary rod for the femoral cuts, extramedullary guide for the tibial cuts, and a spacer block to confirm the appropriate ligament balance, was used by the same surgeon (K.H.). All components were fixed with bone cement. In each patient, it was confirmed that the replaced knee could acquire full extension during the surgery. Patients underwent a standard rehabilitation program, including early range of motion and weight-bearing exercises as tolerated. All patients received prophylactic antibiotics and oral pain killers postoperatively as necessary.

The patients were divided into two groups by age: patients older than 76 years (Group O) and those younger than 75 years (Group Y). Postoperative evaluations including OLS time, KFA, and VAS were done daily from postoperative day 3 to 20 in each group, because the epidural catheter was removed on postoperative day 2.

### Statistical analysis

Multiple regression analysis using the stepwise method was used to identify the factors affecting OLS time. Variables considered in the analysis were OLS time, age, BMI, KFA, FTA, and pain level. OLS time was treated as a dependent variable, and the others were treated as independent variables.

In terms of the age-related recovery process, preoperative measurements were used as controls in each group. Significant differences between the data were evaluated using two-tailed repeated-measures of analysis of variance (ANOVA). After a significant *P* value was found, a post hoc Dunnett test was performed to compare selected mean values. All statistical analyses were done with SPSS® Version 11 for Microsoft Windows (Chicago, IL). *P* values of <0.05 were considered significant in all analyses.

## Results

### Factors affecting the OLS time

Average values of each parameter are presented in Table 1. Mean OLS time was 8.7 s. No patients could perform OLS for more than 60 s. These results demonstrated that patients with knee OA would have poor one-leg standing balance, because the mean OLS time in the normal population (75–79 years old) was 42 s in men and 28 s in women [6].

**Table 1:** Average value of each parameter (mean ± SD)

One-leg standing time	8.9 ± 7.1 s
Age	75.1 ± 3.6
Body mass index	26.8 ± 3.8 kg/m^2^
Knee flexion angle during standing (KFA)	12.5 ± 7.3 Deg.
Femor otibial angle (FTA)	183.7 ± 5.3 Deg.
Visual analogue scale (VAS)	65.8 ± 22.6 mm

Multiple regression analysis using the stepwise method in the current study showed that the coefficient of determination (R2) was 0.38, and the Durbin-Watson ratio was 1.740 (Table 2). According to the stepwise forward regression test, KFA, age, and FTA were entered into this model, while BMI and pain were not entered (Table 2). The partial regression coefficients were: 0.44 for KFA, 0.40 for age, and 0.29 for FTA (Table 3). Therefore, a large KFA during standing, older age, and large FTA were significantly associated with shorter OLS time.

**Table 2:** Results of the multiple regression analysis using the stepwise method

Variables entered in the model:
Age
Knee flexion angle during standing (KFA)
FTA
Variables not entered in the model:
BMI
VAS

**Table 3:** Detailed information of the multiple regression analysis using the stepwise method

	Partial regression coefficient	Standardized partial regression coefficient	*P*	95% confidence interval
KFA	*0.44*	0.09	0.0001	0.22–0.66
Age	*0.40*	0.11	0.0002	0.20–0.59
FTA	*0.29*	0.12	0.047	0.03–0.50

*R* ^2^ = 0.38, Analysis of variance: *P* < 0.0001

Durbin-Watson ratio 1.74

### Age-related recovery process

**Fig. 2:**
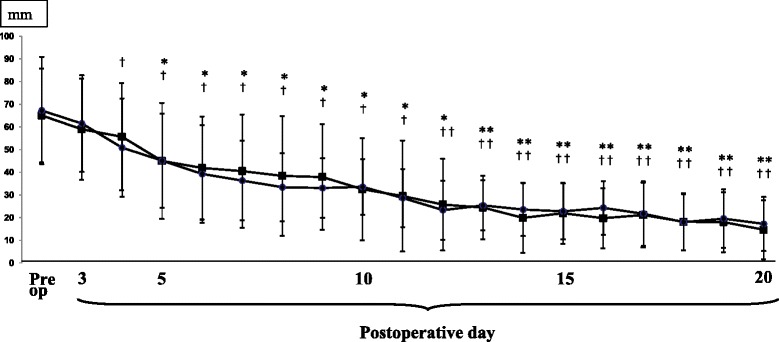
VAS for pain of operated knees during one leg standing. ■: Group O, ●: Group Y (*P < 0.05 and **P < 00.01 in Group O, ^†^ *P* < 0.05, ^††^ *P* < 0.01 in Group Y)

**Fig. 3:**
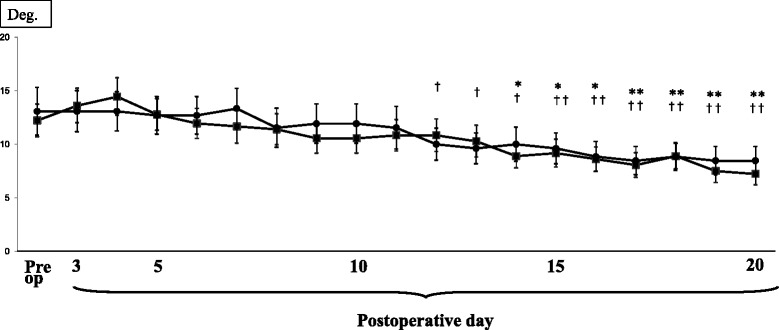
Knee flexion angles of operated knees during one leg standing. *Black filled squares*: Group O. *Black filled circles*: Group Y (*P < 0.05 and **P < 00.01 in Group O, ^†^ *P* < 0.05, ^††^ *P* < 0.01 in Group Y)

**Fig. 4:**
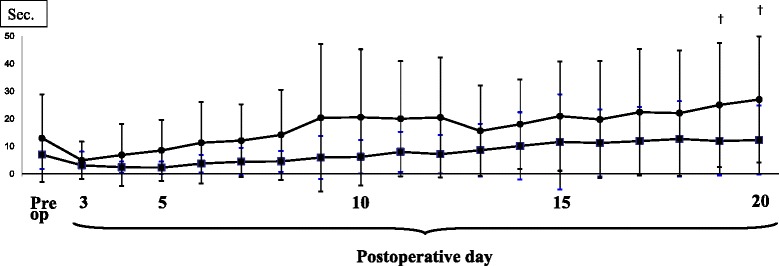
OLS times of operated knees. *Black filled squares*: Group O, *Black filled circles*: Group Y (^†^ *P* < 0.05 in Group Y)

A total of 24 patients (mean age 78 years) were allocated to Group O, and the remaining 16 patients (mean age 68 years) were allocated to Group Y (Table 4). The pain scores are presented in Fig. 2. After TKA, the pain level decreased gradually, and on postoperative day 4 or 5, the pain score was significantly less than the preoperative score in each group. Similar pain reduction was observed in both groups. The KFA values during one-leg standing is shown in Fig. 3. After TKA, subjects could gradually extend the knee, and on postoperative days 12 and 14, the angle during OLS was smaller than the preoperative angle in Group Y and Group O, respectively. Basically, a similar tendency was observed in both groups. The recovery process of OLS time is presented in Fig. 4. After TKA, OLS time was significantly better on postoperative day 19 in Group Y, while significant improvement was not seen in Group O.

**Table 4:** Preoperative patients’ demographics in each group (mean ± SD)

	Group O	Group Y	*P* value^a^
Age	78.3 ± 2.2	68.6 ± 4.2	<0.001
Sex (female/male)	22/2	13/3	0.68
BM (kg/m^2^)I	26 ± 3.8	26.5 ± 3.9	0.92
FTA (Deg.)	184.3 ± 6.8	182.5 ± 4.4	0.43
VAS	65.1 ± 20.7	67.2 ± 23.6	0.78
KFA (Deg.)	12.2 ± 6.5	13.0 ± 8.1	0.74
OLS (s)	6.9 ± 5.2	12.9 ± 14.9	0.14

^a^Values obtained using Mann-Whitney *U* test or Chi square test

## Discussion

Patients with end-stage medial knee OA in the current study had balance impairment during OLS, since the mean OLS time was 8.7 s. Some studies demonstrated that decreased OLS time was related to an increased risk of falls [8, 9]. Basically, falls could lead to osteoporotic fractures, which would result in an increased risk of future functional dependence in patients with knee OA. The results of the current investigation indicated that factors associated with OLS time in patients with end-stage knee OA were the KFA during standing, age, and the FTA. Generally, full knee extension plays an important role in the functional outcome for elderly people. Some previous studies indicated that full knee extension would be important in standing, walking, and stair climbing [17–19]. For example, Cerny et al. investigated the effect of knee flexion contracture on gait using knee flexion contracture simulation and demonstrated that velocity and stride length decreased significantly [17]. Furthermore, the simulated knee flexion contracture would cause mechanical overload of both limbs during gait [18]. The present results support the importance of full extension in patients with knee OA.

On the other hand, both FTA and knee flexion contracture should be corrected by TKA [20], and, thus, TKA would be effective treatment for patients with short OLS time. An understanding of standing balance in patients with end-stage knee OA before and after TKA may be beneficial for assessment of the risk of falling. Particularly in the early postoperative period, ambulation is at its most unsteady [21]. In the present study, similar improvements of subjective pain and extension limitation were seen. In terms of OLS, Cho et al. showed that OLS balance in patients with knee OA improved significantly 11 days after TKA in younger patients [15]. However, OLS time in older patients did not improve for 20 days after TKA. Presumably, a longer rehabilitative period is required in older patients than in younger patients for significant recovery of OLS time. Therefore, the risk of falling still exists for older patients in the early postoperative period after TKA.

Physiological weakness of the quadriceps muscle with age is observed in elderly people [22], and, moreover, knee flexion contracture is frequently seen in patients with end-stage knee OA. Therefore, quadriceps muscle strength exercise or the improvement of knee flexion contracture can be effective treatment to obtain better OLS time in patients with end-stage knee OA [23]. From the current study, to maintain full knee extension in standing or to stand up with the knees extended as much as possible is a key issue for prevention of balance impairment in patients with end-stage knee OA. Furthermore, the risk of falling should be considered particularly in older patients during the early postoperative period after TKA, while TKA can improve OLS time.

Several limitations should be noted in the present study. A weakness of the present study was the inaccuracy in measuring each parameter using analog tools. The inaccuracy of these tools may lead to slight changes in the data, although the general trends would still be evident. Second, subjects in the current investigation were 35 females and 5 males. Although the sex ratio should be close to equal, 85 to 90% of patients with knee OA were females in the clinical situation. It is thus possible that the results of the present study indicate only the trends for female OA patients. However, since the current study is the first to investigate the detailed information of the OLS test in patients with end-stage knee OA and the age-related recovery process following TKA, the results of the present study offer useful information when considering the effect of TKA on OLS time in patients with end-stage OA.

## Conclusions

Factors associated with OLS time in patients with end-stage knee OA were the KFA during standing, age, and the FTA. Even if subjective knee pain and the KFA during OLS were improved by TKA, longer rehabilitation was required for the improvement of OLS time in older patients in the early postoperative period.

## Abbreviations

ANOVA: Analysis of variance
BMI: Body mass index
FTA: Femorotibial angle
KFA: Knee flexion angle
OA: Osteoarthritis
OLS: One-leg standing
TKA: Total knee arthroplasty
VAS: Visual analogue scale
